# Ubiquitination as an Efficient Molecular Strategy Employed in *Salmonella* Infection

**DOI:** 10.3389/fimmu.2014.00558

**Published:** 2014-11-25

**Authors:** Lakshmi A. Narayanan, Mariola J. Edelmann

**Affiliations:** ^1^The Department of Basic Sciences, College of Veterinary Medicine, Mississippi State University, Mississippi State, MS, USA; ^2^Institute for Genomics, Biocomputing and Biotechnology, Mississippi State University, Mississippi State, MS, USA

**Keywords:** ubiquitin, deubiquitinases, E3 ligases, autophagy, *Salmonella enterica* Typhimurium, *Salmonella*-containing vacuole, innate immune response, type III secretion system

## Abstract

The ubiquitin modification has various functions in the host innate immune system in response to the bacterial infection. To counteract the host immunity, *Salmonella* can specifically target ubiquitin pathways by its effector proteins. In this review, we describe the multiple facets of ubiquitin function during infection with *Salmonella enterica* Typhimurium and hypothesize how these studies on the host–pathogen interactions can help to understand the general function of the ubiquitination pathway in the host cell.

## Introduction

*Salmonella enterica* is an intracellular facultative anaerobe that is one of the leading causes of enteric diseases in the United States. Over 2500 serovars belonging to six sub-species of *S. enterica* have been identified. When ingested through contaminated food or water, *Salmonellae* cause disease syndromes such as typhoid, gastroenteritis, bacteremia, and chronic asymptomatic carriage (1, 2). *S. enterica* serovar Typhimurium, the causative agent of gastroenteritis has successfully evolved to cope with host defense mechanisms [reviewed in Ref. (2)]. The roles of different ubiquitin pathways in host innate immune system during *Salmonella* infection are widely recognized and their action involves a wide range of processes – from bacterial sensing to triggering innate immune responses. In retaliation to the host immune responses, bacteria target ubiquitin pathways using several virulence factors [reviewed in Ref. (3)]. In this review, we focus on the impact of ubiquitin pathways during infection with *S. enterica* Typhimurium in the context of the innate immune system. We also highlight how studies on the host–pathogen interactions can help to understand the ubiquitination pathway in the eukaryotic cell.

## Regulation of Innate Immune System in *Salmonella* Infection

To successfully colonize the host, the pathogens battle the highly sophisticated defense mechanisms of the innate and adaptive immune systems. Briefly, after ingestion of *Salmonella*, bacteria encounter the harsh acidic environment of the stomach, which they counteract by inducing the acid tolerance response system (4, 5). In the small intestine, *Salmonellae* are awaited by a thick layer of mucus covering the gut epithelium, while the Paneth cells and epithelial cells in the gastrointestinal tract produce anti-microbial peptides that function by disrupting the bacterial cell membrane integrity, which *Salmonella* can counteract. *Salmonella* is able to invade microfold cells of the Peyer’s patches and non-phagocytic enterocytes, and the internalized bacteria induce membrane ruffling, which causes formation of *Salmonella*-containing vacuole (SCV), an intracellular niche where the bacteria replicate and thrive with the help of bacterial effectors from the Type III Secretion System [T3SS; reviewed in Ref. (2, 6)]. Another line of host defense includes engulfment of *Salmonella* by macrophages, neutrophils, or dendritic cells, which can lead to phagocytosis. On a molecular level, the innate immune system is activated in response to pathogen-associated molecular patterns (PAMPS), which are conserved components detected on the microbes, such as lipopolysaccharides (LPS), peptidoglycan, or lipoteichoic acid. Since these components are physiologically important for bacterial survival, they cannot be altered as an adaptation strategy. PAMPs are recognized by the germline-encoded pattern recognition receptors (PRRs) of the host cells. PRRs are expressed by non-immune and innate immune cells, and include Toll-like receptors (TLRs), NOD-like receptors (NLRs), and RIG-I-like receptors (RLRs) [reviewed in Ref. (7, 8)]. Signals transduced from the PRRs cause activation of transcription factors, e.g., nuclear factor kappa B (NF-κB), or interferon regulatory factors (IRFs). This leads to expression of key cytokines and chemokines that trigger anti-microbial responses and recruit immune cells to the infected area [reviewed in Ref. (9)]. Immune responses have to be ideally and promptly controlled and, therefore, post-translational modifications (PTMs) of proteins, such as ubiquitination, play here a crucial role.

## Ubiquitin – A Small Protein Modifier

Ubiquitination is a PTM characterized by the addition of ubiquitin to a lysine residue of protein substrates. It can mark proteins for degradation or play a non-proteolytic role in regulation of processes such as endocytosis, DNA repair, intracellular trafficking, and signal transduction [reviewed in Ref. (3, 10)]. Ubiquitination is a multi-step process carried out by E1 (ubiquitin-activating enzyme), E2 (ubiquitin-conjugating enzyme), and E3 (ubiquitin ligase) enzymes, and it can be reversed by deubiquitinases (deubiquitinating enzymes). Attachment of a single ubiquitin moiety is called monoubiquitination, which can lead to protein auto-inhibition, and this has been shown for example in ubiquitin receptors involved in endocytosis [reviewed in Ref. (11)]. Apart from that, ubiquitin can form eight distinct chains, in which the C-terminus of a distal moiety is attached to one of the seven lysine residues of ubiquitin (K6, K11, K27, K29, K33, K48, or K63). These distinct ubiquitin chains have different effects on protein substrates. K63-linked chains can affect cell signaling, receptor endocytosis, or processes associated with DNA repair [reviewed in Ref. (12)], and all other ubiquitin chains target proteins for degradation (13). In addition, the polyubiquitin can be linked through the N-terminal M1, and the chains can also have mixed typology. To add to this complexity, there are ubiquitin-like proteins, such as neural precursor cell expressed, developmentally down-regulated 8 (NEDD8), small ubiquitin-related modifier (SUMO), interferon-induced 17 kDa protein (ISG15), autophagy-related (ATG) 8, or ATG12. Since these PTMs have profound effects on protein function, stability, or localization, it is not surprising that they are employed in host responses to bacterial infections, or that bacterial pathogens evolved complex strategies to interrupt normal cell functions and modify these PTMs to their advantage.

## Ubiquitination-Regulated Host Defense Strategies

### *Salmonella* and ubiquitin-regulated selective autophagy

Cells remove unwanted bulk cytosolic materials such as proteins, organelles, or intruding pathogens by autophagy process, which is facilitated by autophagosomes that engulf the cytosolic components and fuse with the lysosomes to form autolysosomes, finally resulting in their degradation. Selective autophagy occurs when the ubiquitin system is used to mark the unnecessary cytosolic materials for degradation via the autophagosomes (3). To selectively bind the ubiquitinated materials, p62, nuclear dot protein 52 kDa (NDP52), and optineurin (OPTN) receptors act as a bridge between the ubiquitinated cargo and the autophagosome (3). *Salmonellae* can be coated with ubiquitin for degradation by the autophagy, but they also developed strategies to escape it. ATG proteins belonging to the ubiquitin or ubiquitin-like families, deubiquitinases, or E3 ligases are described in the sections below (see Table 1).

**Table 1:** **Host proteins relevant in ubiquitin-mediated response to *Salmonella* infection**.

Host protein	Function	Physiological effect	Reference
OPTN	Contains ubiquitin-binding domain; autophagy receptor	Selective autophagy of ubiquitin-coated *Salmonella*	(18)
p62	Autophagy receptor	Autophagy of ubiquitin-coated *Salmonella*	(15, 16)
NDP52	Autophagy receptor	Autophagy of ubiquitin-coated *Salmonella*	(14)
LRSAM1	RING-type E3 ligase	Restriction of bacterial replication, required for autophagy of *Salmonella*	(19)
USP18	ISG15-specific deubiquitinase	Regulation of inflammatory response to *Salmonella*, IFN signaling	(25)
UCH-L1	Deubiquitinase	Increase in bacterial uptake, remodeling of actin cytoskeleton	(26)
HsRMA1	E3 ligase	Ubiquitination of bacterial SopA, induces bacterial escape to cytosol from SCV	(28)
UbcH5c	E2 enzyme	SopB localization to SCV, works with TRAF6	(29)
TRAF6	RING-type E3 ligase	Ubiquitination of bacterial effector SopB, downregulation of SopB activity and its localization in SCV	(29–31)
TRIM21	E3 ligase	Recognition of intracellular antibodies during infection	(27)

#### Function of NDP52, p62, and OPTN in autophagy of *Salmonella*

NDP52 is an autophagy receptor, which is able to detect the ubiquitin moieties on *Salmonella* by using its zinc finger domain. Knockdown of NDP52 leads to enhanced proliferation of bacteria in HeLa cells and to an increase of ubiquitin-coated *Salmonella*. Moreover, NDP52 controls autophagy of *Salmonella* and recruits autophagosomal marker, microtubule-associated protein 1A/1B-light chain 3 [LC3; (14)]. The p62 protein is a ubiquitin-binding protein associated with ubiquitinated protein aggregates that accumulate, for example, in various neurodegenerative disorders (15). In HeLa cells, p62 binds to ubiquitin through its C-terminal ubiquitin-associated (UBA) domain and it also binds to LC3, facilitating autophagy of cytosolic ubiquitin-coated *Salmonella* (16). The autophagy receptor OPTN contains the ubiquitin binding in ABIN and NEMO (UBAN) domain to bind ubiquitin (17) and also binds LC3 through its LIR (LC3-interacting region) motif. OPTN knockdown in HeLa cells during *Salmonella* infection leads to bacterial proliferation. Ubiquitin-binding deficient OPTN mutant or LIR mutant cannot rescue the dysfunction caused by the OPTN knockdown, indicating that both these domains are required to restrict bacterial growth. TANK-binding kinase 1 (Tbk1) phosphorylates OPTN recruited to ubiquitin-coated cytosolic *Salmonella*, thereby enhancing its binding to LC3 and most likely facilitating clearance of cytosolic bacteria through selective autophagy. As mentioned above, there are several *Salmonella*-sensing receptors, including p62, NDP52 as well as OPTN, and all of them bind to ubiquitin-coated *Salmonella*. However, NDP52 and OPTN localize to different microdomains on the surface of ubiquitin-coated *Salmonella* in comparison with p62 (18). This differential recognition might be caused by diverse affinity of these receptors for various ubiquitin linkages or by secondary interactions with other proteins.

#### Host E3 ligase LRSAM1

In a study dissecting the function of autophagy cascade in elimination of *Salmonella*, leucine-rich repeat (LRR) and sterile alpha motif-containing protein 1 (LRSAM1) has been identified as an E3 ubiquitin ligase responsible for recognition and ubiquitination of *Salmonella* and its subsequent autophagy. LRSAM1 co-localizes with *Salmonella*, and a knockdown of LRSAM1 results in increased replication of bacteria in the cytoplasm of HeLa cells. Co-localization of LRSAM1 with *Salmonella* was also observed in infected murine bone marrow-derived macrophages and intra-peritoneal macrophages. LRSAM1 contains a domain commonly found in innate PRRs, LRR, as well as RING domain, which is characteristic of one of the classes of ubiquitin E3 ligases. LRR is required and sufficient for the LRSAM1 localization to *Salmonella*, but RING domain is essential for its ubiquitination. LRSAM1 and previously mentioned NDP52 localize to intracellular bacteria into spatially separated subdomains, but NDP52 recruitment to *Salmonella* is dependent on LRSAM1, which is also required for ubiquitin-associated autophagy and most likely can recognize bacteria by itself. Moreover, polyubiquitination directed by LRSAM1 favors K6- and K27-conjugated ubiquitin chains in comparison to other linkages. LRSAM1, therefore, restricts bacterial replication in the cytoplasm and is crucial for ubiquitin-mediated autophagy (19). This study helped to identify mechanisms and specificity of a novel host ubiquitin E3 ligase and define its function in autophagy. Selective autophagy during *Salmonella* infection is not completely understood; yet, it is clear that it requires ubiquitin pathways to function efficiently and it represents an effective host surveillance mechanism to control *Salmonella* replication and prevent systemic infection.

### Deubiquitination is relevant in inflammasome assembly during *Salmonella* infection

Inflammasome includes PRRs such as NLRs, which are assembled into a multiprotein complex that activates caspase-1 and leads to secretion of proinflammatory interleukins (IL), such as IL-1β, which can lead to pyroptosis, a proinflammatory cell death [reviewed in Ref. (20)]. Role of ubiquitination was investigated in LPS- and *Salmonella*-induced inflammasome. Treatment with the general deubiquitinase inhibitors (PR-619 and WP1130) led to increase in polyubiquitination of NLRP3 (NLR family, pyrin domain containing 3) in N1-8 macrophages stimulated with LPS and ATP. These inhibitors also interfered with caspase-1 activation during *Salmonella* infection (21). This suggests that deubiquitinases are involved in the inflammasome function. Moreover, treatment of cells with b-AP15, which inhibits ubiquitin-specific peptidase 14 (USP14) and ubiquitin carboxy-terminal hydrolase 37 (UCH37), caused inhibition of ATP-, or nigericin- induced IL-1β release from LPS-primed macrophages. Deubiquitinase inhibition also led to impairment in apoptosis-associated speck-like protein containing CARD (ASC) oligomerization without direct inhibition of caspase-1 activity. This has not been shown directly in *Salmonella* infection model and it is not known how these deubiquitinases affect the infection outcome (22). These studies were crucial in identification of a novel mechanism of inflammasome regulation by deubiquitinases.

### ISG15-specific protease important in interferon signaling in *Salmonella* infection

Interferon-induced 17 kDa protein (ISG15) post-translationally modifies other proteins and its expression is induced by type I interferons (IFN) or by exposure of cells to LPS (23). One of the proteins that removes ISG15 modification is ubiquitin-specific peptidase 18 [USP18; (24)]. A mutation in USP18 leads to an increased bacterial load in spleen and liver in mice, and it is also associated with an altered inflammatory response to *Salmonella* infection, e.g., increase in Type 1 IFN or IL-6 secretion, but a decrease in STAT4 phosphorylation and IFN-γ production (25). This suggests that this ISG15-specific deubiquitinase is required for host resistance to *Salmonella* infection by contributing to the IFN signaling, which might also be relevant in other infections.

### UCH-L1 promotes uptake of *Salmonella* in epithelial cells

Ubiquitin C-terminal esterase L1 (UCH-L1) is a deubiquitinase promoting the invasion of cells by *S. enterica* and *Listeria monocytogenes*. The internalization of bacteria by epithelial cells was significantly decreased in UCH-L1 knockdown cells, while the overexpression of UCH-L1 leads to an increased uptake of bacteria. The mechanism, by which this enzyme regulates bacterial uptake possibly involves the actin cytoskeleton remodeling, since overexpression of UCH-L1 was associated with an increase in formation of the actin stress fibers, while overexpression of catalytically inactive C90S mutant of UCH-L1 had an opposite effect (26). This study identified new functions of UCH-L1 in host cells.

### Function of E3 ubiquitin-protein ligase TRIM21 in immune signaling

E3 ubiquitin-protein ligase tripartite motif containing 21 (TRIM21) is a cytosolic antibody receptor that recognizes intracellular antibodies during infection. TRIM21 catalyzes the formation of K63-linked polyubiquitin chains and leads to stimulation of the NF-κB, AP-1, IRF3, IRF5, and IRF7 pathways. During infection of HeLa cells by *Salmonella*, antibodies are carried into the cell by the bacteria. TRIM21 E3 ligase co-localizes to a portion of antibody-bound bacteria. Moreover, antibody-dependent NF-κB signaling is hindered when TRIM21 is knocked down. This study emphasized another general aspect of involvement of ubiquitin in immune signaling (27).

## Exploitation of the Host Responses by *Salmonella*-Encoded Proteins in the Context of Ubiquitin Signaling

*Salmonella* has evolved several defense strategies to survive the hostile environment of the host cell. Since ubiquitin pathway is extensively used by the immune system, bacteria strategically exploit it via their effector proteins. First, SseL and AvrA are deubiquitinases encoded by *Salmonella*, which function by preventing autophagy and inflammatory responses, respectively (Table 2). Second, *Salmonella* effectors SopA, SspH1, SspH2 and Slrp are ubiquitin E3 ligases, which ubiquitinate protein substrates and some are even capable of auto-ubiquitination (Table 2). Third, bacteria take advantage of the host E3 ligases to add ubiquitin moieties to their own proteins, such as SopA, SopB, SopE, or SptP (Table 1). The exploitation of the host responses by *Salmonella*-encoded proteins in the context of ubiquitin signaling is described in the sections below.

**Table 2:** **Bacterial proteins relevant in ubiquitin-mediated response to *Salmonella* infection**.

Bacterial protein	Activity	Substrates	Physiological effect	Host cell type studied	References
SseL	Deubiquitinase	Ubiquitinated aggregates, ALIS	Delayed cytotoxic effect in macrophages, prevention of autophagy	J774 and RAW264.7 macrophages, murine bone marrow-derived macrophages, HeLa	(33–35)
AvrA	Deubiquitinase	IκBα, β-catenin	Inhibition of NF-κB pathway	*In vivo* (mouse), HCT116, HEK293, HeLa	(43)
SopA	E3 ligase	–	–		(46)
SspH2	E3 ligase	Nod1	Modulates innate immunity in host cells by increasing the Nod1-mediated IL-8 secretion	HeLa and HEK293T	(48)
SspH1	E3 ligase	PKN1	Attenuates androgen receptor signaling	HEK293	(52)
Slrp	E3 ligase	Trx	Triggers cell death	HeLa	(54)

### *Salmonella* deubiquitinases

#### *Salmonella* deubiquitinase SseL interferes with autophagy

SseL is *Salmonella*’s effector protein that functions as a deubiquitinase. SseL prevents the autophagy machinery from recognizing ubiquitin aggregates and aggresome-like induced structures (ALIS), which are formed in response to bacterial infection or LPS-treatment (32, 33). Infection with SseL-deficient *Salmonella* strain results in an accumulation of SCV-associated ubiquitinated aggregates in HeLa cells compared to cells infected with wild-type *Salmonella*. Moreover, by deubiquitination of these ubiquitinated aggregates SseL decreases autophagic flux in macrophages and it favors intracellular replication of *Salmonella* in bone marrow-derived macrophages (33). Ubiquitin-driven autophagy was identified as one of the host responses to *Salmonella* and SseL is an effector protein that counteracts this process. Additionally, SseL is necessary for bacterial virulence in mice, required for delayed cytotoxicity by *Salmonella* in macrophages (34, 35), and it binds to the oxysterol-binding protein [OSBP; (36)]. The deubiquitinating activity of SseL is also related to its role in cell lipid metabolism as SseL prevents lipid droplet accumulation in mouse epithelial cells (37). Since lipid droplet metabolism is regulated in autophagy (38), it could potentially be related to SseL’s function in autophagic flux, although the function of SseL appears to be complex and it might involve several substrates and pathways. From the studies on SseL, some more general host mechanisms could be identified, such as the ubiquitin involvement in the selective autophagy.

#### NF-κB pathway modulation by *Salmonella* deubiquitinase AvrA

NF-κB is a conserved family of transcription factors that regulate diverse processes, such as inflammation, immune response, cell growth, and apoptosis [reviewed in Ref. (39, 40)]. Although induction of this pathway provides immediate immune response and host protection, pathogens utilize the immune cells to replicate and spread to other tissues in the host, resulting in systemic infection. For example, infection with the virulent *Salmonella* leads to increased inflammatory response by NF-κB pathway activation, while the avirulent strain has an opposite effect (41). Inhibition of the NF-κB pathway is facilitated by AvrA, which is another *Salmonella*-encoded deubiquitinase (42, 43) that also functions as an acetyltransferase (44). AvrA deubiquitinates and therefore stabilizes IκBα, an inhibitor of NF-κB pathway, thus preventing nuclear translocation of NF-κB p65, which was shown *in vivo* in mice and in epithelial cells. Infection of mice with AvrA-deficient strain of *Salmonella* leads to an increased IκBα degradation and secretion of NF-κB dependent cytokine, IL-6. AvrA also stabilizes an inhibitor of the proinflammatory NF-κB pathway, β-catenin, by preventing its proteasomal degradation via removal of ubiquitin moieties from β-catenin (43). Moreover, AvrA was linked to an increased risk of cancer associated with chronic *Salmonella* infections (45). In summary, AvrA is a bacterial effector protein used to fight the host defense strategies marked by the ubiquitin modification.

### *Salmonella*-encoded ubiquitin E3 ligases

#### SopA E3 ligase controls effective bacterial escape into the cytosol

*Salmonella*’s effector, SopA, is a HECT-like E3 ubiquitin ligase that becomes ubiquitinated by a host E3 ligase HsRMA1 (28, 46), although it is also capable of autoubiquitination (47). In an ubiquitination assay to identify the E2 ligases associated with SopA, UbcH5a, UbcH5c, and UbcH7 were preferentially used by SopA, suggesting a regulatory role in inflammation (46). We discuss effects of SopA polyubiquitination in Section “SopA polyubiquitination regulating bacterial escape.”

#### SspH2 functions in innate immune responses

*Salmonella*’s SspH2 belongs to the novel ubiquitin E3 ligase (NEL) family. It contains LRR domain, which exerts an inhibitory effect on the NEL domain activity, while the NEL domain expressed alone has a 25-fold increase in E3 ligase activity in comparison to a full-length SspH2. Moreover, in epithelial cells, SspH2 increases the Nod1-mediated IL-8 secretion via monoubiquitination, thereby mediating the innate immune response. This function depends on the E3 ubiquitin ligase activity of SspH2 (48). Apart from the identification of a novel bacterial E3 ligase, these studies bring into light a new modification of the host Nod1 protein and its relevance in the IL-8 secretion.

#### Role of SspH1 in androgen receptor signaling

SspH1 is *Salmonella*’s effector protein that is a member of the NEL family of ubiquitin E3 ligases (49). It is capable of ubiquitination of protein kinase N1 (PKN1), which functions in androgen receptor (AR) signaling (50, 51). The wild-type SspH1 ubiquitinates PKN1 when co-expressed in HEK293 cells and targets it to the 26S proteasome for degradation, but a catalytic mutant of SspH1 lacks this function. Attenuation of AR activation was observed when wild-type SspH1 was transiently expressed in HEK293 cells in comparison to expression of C492A catalytic mutant or PKN1-interaction mutant. By mediation of ubiquitination and subsequent degradation of PKN1, SspH1 attenuates AR signaling, which might be important in regulation of the cellular immunity during *Salmonella* infection (52, 53).

#### Slrp ubiquitinates Trx and has cytotoxic effect on host cells

Slrp is another *Salmonella*’s ubiquitin E3 ligase, which belongs to NEL E3 ligase family, and it also contains LRR domains. Mammalian thioredoxin-1 (Trx) is as a binding partner of Slrp (54). Trx proteins regulate redox-related signaling, synthesis of cytokines, growth, and apoptosis [reviewed in Ref. (55, 56)]. Slrp ubiquitinates Trx, while mutation in the cysteine active site of Slrp (C546) disables this activity. In HeLa cells, Slrp expression results in reduction of Trx’s activity in confluent but not in growing cultures. *Salmonella* infection of HeLa cells decreases Trx activity, which also correlates with an increase in cell death. Collectively, these findings suggest that the E3 ligase activity of Slrp is partially responsible for the cytotoxic effect on HeLa cells during infection (54).

### Ubiquitination of bacterial effector proteins

*Salmonella*’s effector proteins, SopA, SopB, SopE, and SptP, have been all shown to be ubiquitinated, and in some cases, it marks them for proteasome-dependent degradation [e.g., SopE and SptP; (57)]. SopA and SopB ubiquitination is relatively well understood and it is described below.

#### SopA polyubiquitination regulating bacterial escape

SopA is an ubiquitin E3 ligase that can be ubiquitinated by a host E3 enzyme HsRMA1 (28, 46). SopA ubiquitination by HsRMA1 is important in regulation of the bacterial escape from SCV, as shown by using HsRMA1 knockdown study. Also, compared to the wild-type *Salmonella*, a *sopA Salmonella* mutant has an impaired ability to escape from SCV into the cytosol in HeLa cells. This together demonstrates that ubiquitination of this effector protein is important for an effective bacterial escape into the cytosol and that host E3 ligase HsRMA1 contributes to this function (28).

#### Function of SopB polyubiquitination affects its activity and intracellular localization

*Salmonella*’s SopB, is a phosphoinositide phosphatase that regulates several physiological processes owing to its phosphatase activity [reviewed in Ref. (58)]. It is ubiquitinated by host E3 ligase TRAF6 and E2 enzyme UbcH5c (29). Interestingly, SopB ubiquitination does not affect SopB protein stability but it downregulates SopB activity at the plasma membrane. SopB ubiquitination also leads to an internalization of SopB into the host cells, and it causes retention of SopB in the SCV (31). Ubiquitination of SopB is essential for SopB-dependent recruitment of Rab5 to SCV (30), but not for PI(3)P generation on the SCV (31). In summary, SopB ubiquitination by the host ubiquitin machinery is not related to its stability but it does regulate its enzymatic activity at the plasma membrane as well as its intracellular localization.

## Conclusion

Ubiquitination is a widespread PTM critical in regulation of many host cellular pathways. However, due to the expansive involvement of this modification in cellular processes, a lot has to be learnt about the function and mechanisms of ubiquitination. Since *Salmonella* infection is important from the human health point of view, there are many efforts concentrated on dissecting the cellular responses to this bacterial infection. In particular, the involvement of ubiquitination in the host–pathogen interactions during *Salmonella* infection is extensive. Due to the work on ubiquitin pathways in *Salmonella* infection, functions and substrates of such deubiquitinating enzymes as USP18 (25) and UCH-L1 (26) were identified. Similarly, more functions were discovered of the host ubiquitin E3 ligases, such as HsRMA1 (28), LRSAM1 (19), and TRAF6, and about host ubiquitin E2 enzyme, UbcH5c (29). Furthermore, the work focused on autophagic clearance of *Salmonella* has been critical in identification of novel mechanisms controlling the autophagy receptors. Specifically, TBK1-mediated phosphorylation of a receptor OPTN leads to selective autophagy of ubiquitin-coated *Salmonella*. This can constitute a more universal mechanism for selective autophagy (18). In fact, TBK1-mediated phosphorylation of OPTN has been recently shown to regulate autophagic clearance, which is relevant in autophagy-mediated degradation of misfolded protein inclusions, for example, in some neurodegenerative disorders (59). All these examples highlight how studies on *Salmonella* infection can lead to characterization of general mechanisms in host cells, and to a better understanding of ubiquitin enzymes that have physiological roles beyond the responses to the bacterial infection.

## Conflict of Interest Statement

The authors declare that the research was conducted in the absence of any commercial or financial relationships that could be construed as a potential conflict of interest.
